# Emergence and Genetic Characteristics of H5N1, H5N6, and H5N3 Clade 2.3.4.4b High Pathogenicity Avian Influenza Viruses in South Korea During the 2023–2024 and 2024–2025 Winter Seasons

**DOI:** 10.1155/tbed/8053623

**Published:** 2026-02-24

**Authors:** Ra Mi Cha, Yunyueng Jang, Min-Ji Park, Jinmyeung Kim, Jong-Min Kim, Eui Hyeon Lim, Gyeong-Beom Heo, Se-Hee An, Kwang-Nyeong Lee, Youn-Jeong Lee, Eun-Kyoung Lee

**Affiliations:** ^1^ Avian Influenza Research and Diagnostic Division, Animal and Plant Quarantine Agency, 177 Hyeoksin 8-ro, Gimcheon-si, 39660, Gyeongsangbuk-do, Republic of Korea, qia.go.kr

**Keywords:** H5N1, H5N3, H5N6, genotype, high pathogenicity avian influenza, phylogenetic analysis

## Abstract

Since 2020, clade 2.3.4.4b H5Nx high pathogenicity avian influenza viruses (HPAIVs) have consistently spread across continents, causing outbreaks worldwide. During the 2023–2024 (23/24) and 2024–2025 (24/25) winter seasons, clade 2.3.4.4b H5Nx HPAIVs caused multiple outbreaks on poultry farms and were detected in wild birds in South Korea. In this study, we present the emergence of clade 2.3.4.4b H5N1, H5N6, and H5N3 HPAIVs and examine the genetic characteristics of these viruses isolated from poultry and wild birds during the 23/24 and 24/25 winter seasons. In the 23/24 season, H5N6 and H5N1 HPAIVs caused outbreaks (poultry: 32, wild bird: 19), whereas in the 24/25 season, H5N3 and H5N1 HPAIVs were detected (poultry: 47 and wild bird: 43). Notably, a novel H5N3 HPAIV was isolated from northern pintail and identified as a reassortant virus distinct from the previously reported H5N3 HPAIV. Phylogenetic analysis of the hemagglutinin (HA) gene showed that viruses from both seasons were closely related to HPAIVs isolated in Eurasia. Gene constellation analysis identified two distinct genotypes of H5N1 viruses (23G0‐G1) and one genotype of H5N6 virus (23G2) during the 23/24 season, with the latter being the dominant subtype in that period. In the 24/25 season, five distinct genotypes of H5N1 viruses (24G0–G4) were detected, with 24G1 being the major circulating genotype. Our results suggest that H5N6 HPAIVs appear to be circulating in East Asia alongside H5N1 viruses. Furthermore, in the 24/25 season, two genotypes of H5N1 viruses identified in South Korea were also detected in neighboring countries during the same period: 24G0 was detected in both Korea and Japan, while 24G1 was detected in Korea, Japan, and Mongolia. Therefore, a better understanding of the genetic characteristics of HPAIVs is important for inferring virus introduction and implementing effective control measures in the field.

## 1. Introduction

H5Nx high pathogenicity avian influenza viruses (HPAIVs) originating from the A/Goose/Guangdong/1/1996 (Gs/Gd) lineage have been disseminated worldwide and have caused outbreaks in both domestic and wild birds, resulting in huge economic losses and posing persistent threats to public health [1–6]. Gs/Gd H5 HPAIVs were known to have undergone extensive reassortment events, resulting in diverse hemagglutinin (HA) phylogenetic clades and H5Nx subtypes.

Since 2020, clade 2.3.4.4b H5Nx HPAIVs have demonstrated consistent intercontinental spread and caused global outbreaks [6–9]. During the 2023–2024 (23/24) and 2024–2025 (24/25) seasons, a relatively lower number of HPAI virus detections in poultry and wild birds were reported in Europe compared to the major H5N1 HPAI epidemic in 2022–2023 with mass mortality in seabirds [10–13]. In America, after H5N1 HPAI was introduced in late 2021, the virus caused sustained outbreaks in poultry and wild birds. The virus eventually spilled over into dairy cattle in the USA in 2024 and also caused large‐scale die‐offs of seabirds and marine mammals in South America [4, 5, 14–16]. Moreover, human infections with 2.3.4.4b H5Nx HPAI viruses in multiple countries have been reported due to the spread of H5Nx HPAI outbreaks, posing increased public health concern [6, 17].

Since 2021, South Korea has experienced epidemic outbreaks of clade 2.3.4.4b H5 HPAIVs, which are mostly correlated with global outbreaks. In the 2022–2023 winter season, H5N1 HPAIVs caused multiple outbreaks in poultry farms and wild birds. Phylogenetic analysis of the HA gene revealed that South Korean H5N1 HPAIV isolates were closely related to Eurasian and American HPAIVs isolated in the 2022–2023 season [18]. In addition, 21 distinct genotypes were identified among the H5N1 viruses, demonstrating substantial genetic diversity similar to that observed in Japan and Europe during the 2022–2023 season [11, 19]. In the 23/24 winter season, clade 2.3.4.4b H5N6 and H5N1 HPAIVs were detected in a wild bird and at broiler duck farms in December 2023 [20], and both subtypes caused outbreaks in domestic poultry. In the following 24/25 winter season, a novel H5N3 HPAIV was first identified in northern pintail in October 2024; however, multiple outbreaks in poultry and wild birds were caused by H5N1 HPAIVs. In this study, we report the emergence of clade 2.3.4.4b H5N1, H5N6, and H5N3 HPAIVs and investigate the genetic characteristics of these viruses isolated from poultry in South Korea during the 23/24 and 24/25 winter seasons.

## 2. Materials and Methods

### 2.1. Viruses

In the 23/24 winter season, clade 2.3.4.4b H5N6 and H5N1 HPAIVs were isolated from a total of 32 outbreaks in poultry farms (H5N6/N1, *n* = 1; H5N6, *n* = 25; H5N1, *n* = 6) by the Animal and Plant Quarantine Agency (APQA) (Supporting Information 1: Table S1) and from 19 wild bird cases (H5N6, *n* = 8; H5N1, *n* = 11) detected by the APQA (*n* = 4) and the National Institute of Wild Disease Control and Prevention (NIWDC) (*n* = 15) [21, 22]. In the 24/25 winter season, 47 outbreaks in poultry farms were caused by H5N1 HPAIVs from October 2024 to April 2025. In wild birds, a total of 43 HPAI cases (H5N3, *n* = 1; H5N1, *n* = 42) were detected by the APQA (*n* = 5) and NIWDC (*n* = 38) [23] (https://me.go.kr/niwdc/web/index.do?menuId=50). Notably, a novel H5N3 HPAIV strain was isolated from a northern pintail on October 2, 2024. In total, 89 H5N1, H5N6, and H5N3 HPAIVs isolated during the 23/24 and 24/25 seasons by the APQA were genetically characterized (including two virus isolates [H5N1/H5N6] from one farm outbreak in the 23/24 season [20]). In addition, wild bird species were identified using a DNA barcoding system using mitochondrial DNA from fecal samples, as previously described [24].

### 2.2. Sequencing, Phylogenetic Analysis, and Genome Constellation

H5Nx HPAIV isolates were sequenced as previously described [18, 25, 26]. Briefly, viral RNA from HPAIV isolates was extracted using a Patho Gene‐spin DNA/RNA Extraction Kit (iNtRON Biotechnology, Seoul, South Korea), following the manufacturer’s instructions. Reverse transcription and polymerase chain reactions (RT‐PCRs) were performed using the Superscript III First‐Strand Synthesis System (Invitrogen, MA, USA) and the AccuPrime Taq DNA Polymerase system (Invitrogen) using a previously described primer set [27]. The sequencing library was prepared from the purified PCR products using the Nextera DNA Flex Library Prep Kit (Illumina, San Diego, CA, USA), and whole genome sequencing was conducted using the MiSeq next‐generation sequencing platform (Illumina, San Diego, CA, USA). Genome sequences were assembled and analyzed using BioEdit software and CLC Genomics Workbench (Qiagen, Germantown, MD, USA). The final HPAIV sequences obtained in this study have been deposited in the Global Initiative on Sharing All Influenza Data (GISAID) database (http://platform.gisaid.org) (accession numbers listed in Supporting Information 1: Table S1).

Phylogenetic analyses were performed on HA and neuraminidase (NA) genes of H5Nx HPAIV isolates. Reference datasets were constructed for each gene using sequences obtained from GISAID (https://www.gisaid.org/) (Supporting Information 2: Table S3). For the HA gene, we performed maximum clade credibility (MCC) tree analysis as described below, and for the NA genes, maximum‐likelihood phylogenetic analysis was performed by MEGA version 6.0 with 1000 bootstrap iterations and high bootstrap support (>70%). The genome constellation analysis was performed as previously described [18]. Briefly, a phylogenetic tree analysis was conducted using RAxML version 8.2.12 with a gamma distribution and a general time‐reversible model [28]. A dataset was constructed for each internal gene, including PB2, PB1, PA, NP, MP, and NS, using all avian influenza virus sequences collected from January 1, 2015, to February 28, 2025 (accessed on March 25, 2025) and obtained from the GISAID and GenBank databases. Next, the datasets were reduced using the Cluster Database at High Identity with Tolerance (CD‐HIT) suite and used for phylogenetic analysis (Figure S6). Genotypes were classified according to the phylogenetic tree topology, and a nucleotide sequence identity > 97% was considered significant when the bootstrap support value exceeded 70%.

### 2.3. MCC Tree Analysis

MCC tree analysis was performed to analyze the HA genes of Korean H5Nx HPAIVs using Bayesian evolutionary analysis by sampling trees (BEAST) software, version 1.10.4, as previously described, with modifications [29]. The dataset included the HA genes of viral isolates from poultry (chickens and ducks) farms and wild birds during the 23/24 and 24/25 seasons. An uncorrelated log‐normal relaxed clock model, the Hasegawa–Kishino–Yano nucleotide substitution model, and the Gaussian Markov random field Bayesian Skyride coalescent prior were used for the MCC tree analysis; three posterior tree files with effective sample sizes > 200 in Tracer version 1.7.1 (http://tree.bio.ed.ac.uk/software/tracer/) after 50 million generations were combined using LogCombiner version 1.10.4 (https://www.beast2.org/programs/). A burn‐in of 10% was applied to each run, and MCC trees were generated using TreeAnnotator version 1.8.1 (http://beast.bio.ed.ac.uk/TreeAnnotator/) and visualized using FigTree version 1.4.4 (http://tree.bio.ed.ac.uk/). The time to the most recent common ancestor (tMRCA) was estimated based on the height of the common ancestor nodes.

## 3. Results and Discussion

### 3.1. HPAIVs Isolated in the 2023–2024 and 2024–2025 Winter Seasons

In the 23/24 winter season, clade 2.3.4.4b H5N6 and H5N1 HPAIVs were introduced into South Korea in wild birds and domestic poultry [20]. From December 2023 to May 2024, 32 outbreaks were reported in poultry farms (chickens, *n* = 17; ducks, *n* = 15) (Supporting Information 1: Figure S1; Korean Animal Health Integrated System). In addition, 19 HPAI cases were detected in wild birds by the APQA (*n* = 4) and the NIWDC (*n* = 15) (Supporting Information 1: Figure S1) [21, 22]. In the 2024–2025 winter season, a novel clade 2.3.4.4b H5N3 HPAIV was first detected in fecal samples collected from northern pintail in the Mangyeong River Basin in Gunsan, Jeollabuk‐do (latitude: 35°52′ 18″ N; longitude 126°41′ 9″ E), South Korea on October 2, 2024 (Supporting Information 1: Table S1). However, after this single H5N3 detection, H5N1 HPAIV emerged as the major subtype, causing 47 outbreaks in poultry farms (chickens, *n* = 29; ducks, *n* = 18) from October 2024 to April 2025. In wild birds, a total of 43 HPAI cases were detected by the APQA (*n* = 5) and the NIWDC (*n* = 38) [23] (Supporting Information 1: Figure S1; Korean Animal Health Integrated System).

There were fewer HPAIV outbreaks in Korea during the 23/24 and 24/25 seasons than during the previous 2022–2023 winter season [18]. However, most HPAI outbreaks were observed in the western regions of South Korea, which is consistent with previous outbreaks, as these regions are well‐known habitats for migratory wild birds and have high poultry population [25, 30] (Supporting Information 1: Figure S2). Interestingly, during the 23/24 season, two subtypes (H5N1 and H5N6) cocirculated in Korea, and one poultry farm was confirmed to have concurrent infections with both subtypes [20]. Although H5N1 was the dominant subtype in global circulation during 2023–2024, H5N6 was the major HPAIV subtype in South Korea during the 23/24 seasons and neighboring countries, such as Japan and China, have also reported the presence of H5N6 HPAIV at around the same time [31–34]. These results may suggest that the H5N6 subtype was the regionally circulating HPAIV strain in East Asia. However, in the 24/25 season, the major subtype causing most outbreaks in South Korea was H5N1, which correlated with global H5 HPAI circulation. We believe that during the 23/24 winter season, H5N6 HPAIVs circulated among migrating wild birds along certain flyways for unknown reasons, affecting East Asia and resulting in the cocirculation of H5N6 and H5N1 in South Korea. However, during the 24/25 season, the globally circulating H5N1 HPAIV was dominant in wild birds, consequently affecting South Korea. In addition, in the 24/25 season, a novel H5N3 HPAIV was first detected in a northern pintail, a typical migratory wild bird found in South Korea.

### 3.2. Genetic Characterization of H5 HPAIV Isolates

**Figure 1:**
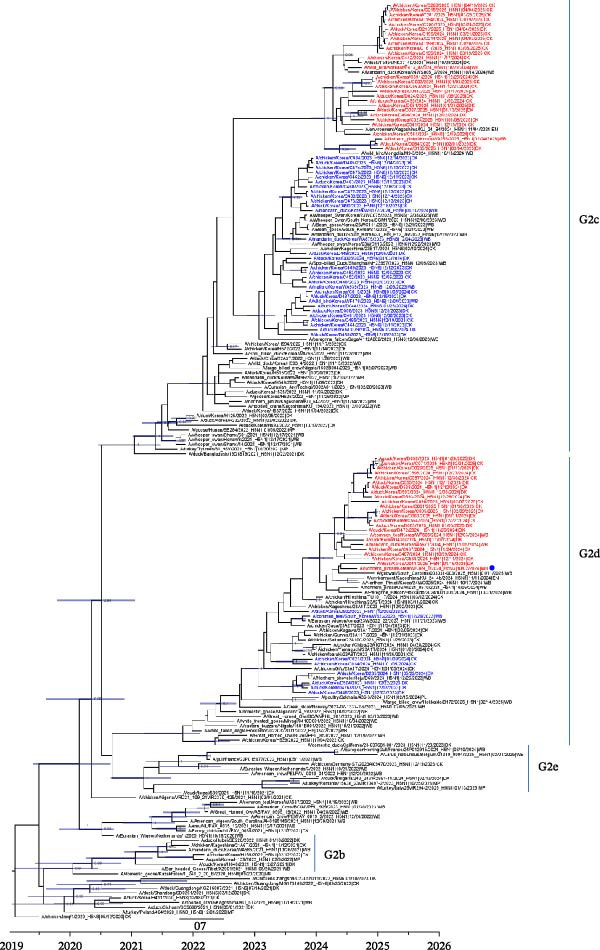
Maximum clade credibility (MCC) tree of the hemagglutinin (HA) gene of Korean H5N1, H5N6, and H5N3 HPAI virus isolates from 2023 to 2025. The scale bar represents the number of nucleotide substitutions per site, and a monophyletic cluster was defined when bootstrap values (1000 replicates) were greater than 70%. HPAIVs isolated from poultry and wild birds during the 23/24 winter season are shown in blue, and those from the 24/25 winter season are shown in red. All viruses highlighted in color were isolated in APQA. Blue dot indicates a novel H5N3 HPAIV isolated in Korea.

The HA genes of HPAIVs from the 23/24 and 24/25 seasons were analyzed using MCC tree analysis. Total 89 South Korean H5N1 virus isolates from poultry (*n* = 79) and wild birds (*n* = 10) were analyzed (Supporting Information 1: Table S1). All HPAIV isolates were confirmed to belong to clade 2.3.4.4b and had multiple basic amino acid sequences (PLRERRKR ^∗^GLF) at the HA cleavage site (Supporting Information 1: Table S1), indicating high pathogenicity. According to the HA subgroup system used in previous studies [18, 19, 31, 35], the HA genes of Korean HPAIVs clustered into both the G2c and G2d subgroups (Figure 1), differing from the 2022–2023 season, in which the G2c subgroup was the most dominant [18]. Notably, the HA and N6 genes of 23/24 H5N6 viruses were closely related to H5N6 HPAIVs isolated in East Asia, including China (A/spot‐billed duck/Shanghai/NH23997/2023(H5N6)) and Japan (A/peregrine falcon/Saga/4112A002/2023(H5N6)) during a similar period (Figure 1, S5). These findings are consistent with a previous report [32] and further indicate that H5N6 HPAIVs may have circulated in East Asia during the 23/24 winter season [31, 33, 34]. In addition, in 24/25 H5N1 viruses, the H5 (G2c and G2d) and N1 genes showed close relationships with HPAIVs isolated in Eurasia, including Japan and Mongolia (Figure 1, Supporting Information 1: Figure S2) [35]. Molecular dating analyses showed that the tMRCA of the 23/24 season (G2c group) was estimated to be August 5, 2023 (95% Bayesian credible interval (BCI): May 26, 2023–October 8, 2023) with the G2d group showing a comparable estimate. For the 24/25 seasons, the G2c group tMRCA was June 9, 2024 (95% BCI: April 9, 2024–August 11, 2024), and the G2d group also exhibited a similar estimate (Supporting Information 1: Figure S4). These results suggest that the 23/24 and 24/25 HPAI viruses may be descendants of H5Nx viruses from the spring‐summer of each year, which correlated with previous reports [23, 32]. Moreover, amino acid sequence analysis of H5 HPAIVs showed that all H5 isolates lacked critical mutations in mammalian adaptive AA markers, such as E627K and D701N in PB2 and Q226L and G228S in HA (data not shown), in agreement with earlier studies [20, 23].

### 3.3. Genome Constellation of HPAIVs Isolated in South Korea

The genome constellations of Korean HPAIVs were analyzed through phylogenetic analysis of each gene segment. The HA genes of 23/24 and 24/25 Korean HPAIVs were classified as a single H5 group based on the genotyping criteria (>97% nucleotide similarity). However, two subgroups (G2c and G2d) of HA genes appeared to circulate in Korea during 2023–2025 (Figure 1).

In the 23/24 season, two distinct genotypes of H5N1 viruses (23G0–G1) and a single genotype of H5N6 virus (23G2) were detected (Figure 2A, Supporting Information 1: Figure S7, Table 1). Because the H5N6 virus was the major subtype during the 23/24 season, instead of multiple genotype introductions, cocirculation of H5N1 and H5N6 was observed for a relatively short 3‐month period (December, 2023–February, 2024) (Figure 2A). In addition, previous studies from Japan and China showed that H5N6 isolates were genetically similar to H5N6 viruses from Korea, confirming that recombinant H5N6 viruses might have been introduced by migratory birds [31, 32] via multiple pathways and suggesting regional H5N6 HPAIV circulation in East Asia in 2023 (Figure 3A).

Figure 2Gene constellation of South Korean H5N1 and H5N6 HPAIVs isolated weekly from poultry farms and wild birds during 2023–2025. The genotypes of the H5Nx HPAIV isolates are shown according to their isolation date. In the 2023–2024 winter season, two distinct genotypes of H5N1 viruses (23G0–23G1) and one genotype of H5N6 viruses (23G2) were identified (A). In the 2024–2025 winter season, five distinct genotypes of H5N1 viruses (24G0–24G4) were detected (B).

**Figure:**
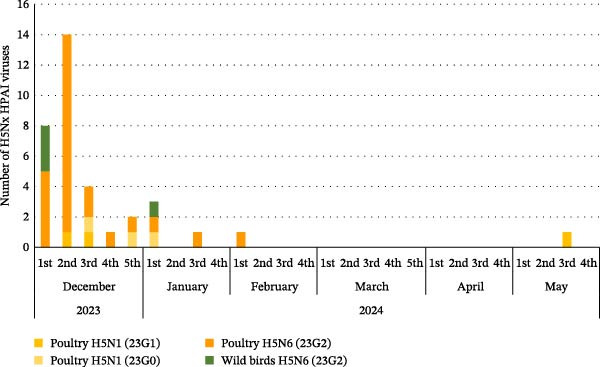
(A)

**Figure:**
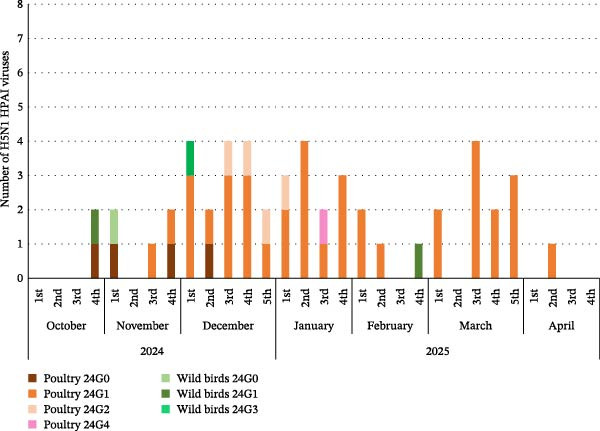
(B)

Figure 3Geographical distribution of genotypes of clade 2.3.4.4b HPAIVs detected during 2023–2025: H5N1 and H5N6 HPAIVs in 23/24 winter season (A) and H5N1 HPAIV in the 24/25 winter season (B). Gene segments were indicated by the eight horizontal bars (top to bottom): PB2, PB1, PA, HA, NP, NA, M, and NS.

**Figure:**
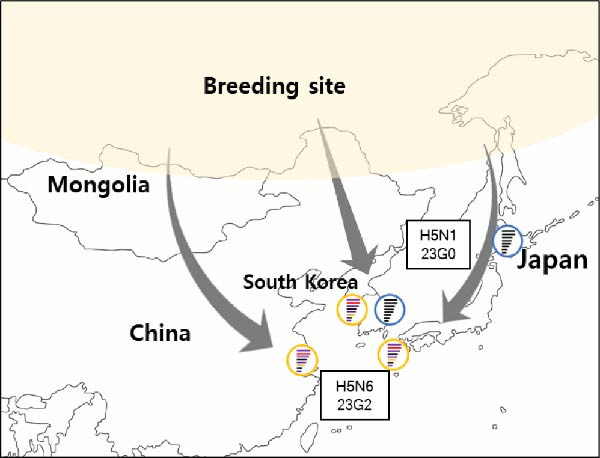
(A)

**Figure:**
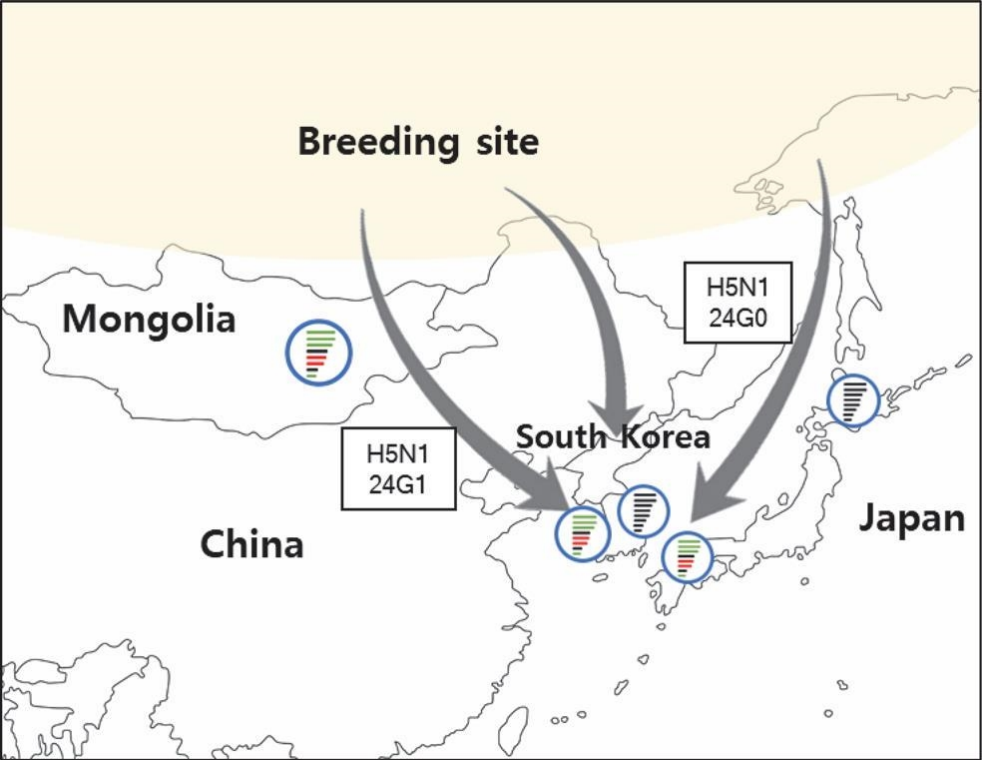
(B)

**Table 1:** Genome constellation of H5Nx (clade 2.3.4.4b) HPAI in South Korea in 2023/2024 and 2024/2025 winter season.

Season	Host	Virus name	Subtype	Reassortant name	Genotype	Phylogenetic group within each gene segment							
						PB2	PB1	PA	HA	NP	NA	MP	NS
2t3/24 H5N1	Poultry	D448‐N1/23	H5N1	AAA5A1AA	23G0	A	A	A	H5	A	N1	A	A
		D476/23	H5N1	CCC5D1AB	23G1	C	C	C	H5	D	N1	A	B
		D502/23	H5N1	AAA5A1AA	23G0	A	A	A	H5	A	N1	A	A
		D504/23	H5N1	CCC5D1AB	23G1	C	C	C	H5	D	N1	A	B
		C014/24	H5N1	AAA5A1AA	23G0	A	A	A	H5	A	N1	A	A
		C021/24	H5N1	AAA5A1AA	23G0	A	A	A	H5	A	N1	A	A
		D285/24	H5N1	CCC5D1AB	23G1	C	C	C	H5	D	N1	A	B

23/24 H5N6	Wild birds	WA859/23	H5N6	CCC5D6AB	23G2	C	C	C	H5	D	N6	A	B
		WA875/23	H5N6	CCC5D6AB	23G2	C	C	C	H5	D	N6	A	B
		WF478/23	H5N6	CCC5D6AB	23G2	C	C	C	H5	D	N6	A	B
		WA939/23	H5N6	CCC5D6AB	23G2	C	C	C	H5	D	N6	A	B
		WA944/23	H5N6	CCC5D6AB	23G2	C	C	C	H5	D	N6	A	B
		WB172/24	H5N6	CCC5D6AB	23G2	C	C	C	H5	D	N6	A	B
	Poultry	D448‐N6/23	H5N6	CCC5D6AB	23G2	C	C	C	H5	D	N6	A	B
		D449/23	H5N6	CCC5D6AB	23G2	C	C	C	H5	D	N6	A	B
		C452/23	H5N6	CCC5D6AB	23G2	C	C	C	H5	D	N6	A	B
		C453/23	H5N6	CCC5D6AB	23G2	C	C	C	H5	D	N6	A	B
		C460/23	H5N6	CCC5D6AB	23G2	C	C	C	H5	D	N6	A	B
		C461/23	H5N6	CCC5D6AB	23G2	C	C	C	H5	D	N6	A	B
		C462/23	H5N6	CCC5D6AB	23G2	C	C	C	H5	D	N6	A	B
		C464/23	H5N6	CCC5D6AB	23G2	C	C	C	H5	D	N6	A	B
		D463/23	H5N6	CCC5D6AB	23G2	C	C	C	H5	D	N6	A	B
		C469/23	H5N6	CCC5D6AB	23G2	C	C	C	H5	D	N6	A	B
		C472/23	H5N6	CCC5D6AB	23G2	C	C	C	H5	D	N6	A	B
		C473/23	H5N6	CCC5D6AB	23G2	C	C	C	H5	D	N6	A	B
		C474/23	H5N6	CCC5D6AB	23G2	C	C	C	H5	D	N6	A	B
		C475/23	H5N6	CCC5D6AB	23G2	C	C	C	H5	D	N6	A	B
		C483/23	H5N6	CCC5D6AB	23G2	C	C	C	H5	D	N6	A	B
		C484/23	H5N6	CCC5D6AB	23G2	C	C	C	H5	D	N6	A	B
		D485/23	H5N6	CCC5D6AB	23G2	C	C	C	H5	D	N6	A	B
		D487/23	H5N6	CCC5D6AB	23G2	C	C	C	H5	D	N6	A	B
		D488/23	H5N6	CCC5D6AB	23G2	C	C	C	H5	D	N6	A	B
		D490/23	H5N6	CCC5D6AB	23G2	C	C	C	H5	D	N6	A	B
		C495/23	H5N6	CCC5D6AB	23G2	C	C	C	H5	D	N6	A	B
		D508/23	H5N6	CCC5D6AB	23G2	C	C	C	H5	D	N6	A	B
		D005/24	H5N6	CCC5D6AB	23G2	C	C	C	H5	D	N6	A	B
		C015/24	H5N6	CCC5D6AB	23G2	C	C	C	H5	D	N6	A	B
		D044/24	H5N6	CCC5D6AB	23G2	C	C	C	H5	D	N6	A	B
		D079/24	H5N6	CCC5D6AB	23G2	C	C	C	H5	D	N6	A	B

24/25 H5N1	Wild birds	WF369‐1/24	H5N3	AAA5A3AA	H5N3 (24G0 + N3)	A	A	A	H5	A	N3	A	A
		WF413‐4/24	H5N1	BBB5B1AB	24G1	B	B	B	H5	B	N1	A	B
		WA671/24	H5N1	AAA5A1AA	24G0	A	A	A	H5	A	N1	A	A
		WF505/24	H5N1	EAE5F1AC	24G3	E	A	E	H5	F	N1	A	C
		WA255/25	H5N1	BBB5B1AB	24G1	B	B	B	H5	B	N1	A	B
	Poultry	C407/24	H5N1	AAA5A1AA	24G0	A	A	A	H5	A	N1	A	A
		D430/24	H5N1	AAA5A1AA	24G0	A	A	A	H5	A	N1	A	A
		C442/24	H5N1	BBB5B1AB	24G1	B	B	B	H5	B	N1	A	B
		C467/24	H5N1	AAA5A1AA	24G0	A	A	A	H5	A	N1	A	A
		D472/24	H5N1	BBB5B1AB	24G1	B	B	B	H5	B	N1	A	B
		C495/24	H5N1	BBB5B1AB	24G1	B	B	B	H5	B	N1	A	B
		D494/24	H5N1	BBB5B1AB	24G1	B	B	B	H5	B	N1	A	B
		D503/24	H5N1	BBB5B1AB	24G1	B	B	B	H5	B	N1	A	B
		C534/24	H5N1	AAA5A1AA	24G0	A	A	A	H5	A	N1	A	A
		D538/24	H5N1	BBB5B1AB	24G1	B	B	B	H5	B	N1	A	B
		C541/24	H5N1	BBB5B1AB	24G1	B	B	B	H5	B	N1	A	B
		D555/24	H5N1	BBB5B1AB	24G1	B	B	B	H5	B	N1	A	B
		C561/24	H5N1	BBB5C1AB	24G2	B	B	B	H5	C	N1	A	B
		C563/24	H5N1	BBB5B1AB	24G1	B	B	B	H5	B	N1	A	B
		C564/24	H5N1	BBB5B1AB	24G1	B	B	B	H5	B	N1	A	B
		C591/24	H5N1	BBB5C1AB	24G2	B	B	B	H5	C	N1	A	B
		D593/24	H5N1	BBB5B1AB	24G1	B	B	B	H5	B	N1	A	B
		C595/24	H5N1	BBB5B1AB	24G1	B	B	B	H5	B	N1	A	B
		C597/24	H5N1	BBB5B1AB	24G1	B	B	B	H5	B	N1	A	B
		C003/25	H5N1	BBB5C1AB	24G2	B	B	B	H5	C	N1	A	B
		D005/25	H5N1	BBB5B1AB	24G1	B	B	B	H5	B	N1	A	B
		C023/25	H5N1	BBB5B1AB	24G1	B	B	B	H5	B	N1	A	B
		D024/25	H5N1	BBB5C1AB	24G2	B	B	B	H5	C	N1	A	B
		C026/25	H5N1	BBB5B1AB	24G1	B	B	B	H5	B	N1	A	B
		D027/25	H5N1	BBB5B1AB	24G1	B	B	B	H5	B	N1	A	B
		D041/25	H5N1	BBB5B1AB	24G1	B	B	B	H5	B	N1	A	B
		D044/25	H5N1	BBB5B1AB	24G1	B	B	B	H5	B	N1	A	B
		D051/25	H5N1	DDD5E1AB	24G4	D	D	D	H5	E	N1	A	B
		D058/25	H5N1	BBB5B1AB	24G1	B	B	B	H5	B	N1	A	B
		C061/25	H5N1	BBB5B1AB	24G1	B	B	B	H5	B	N1	A	B
		D063/25	H5N1	BBB5B1AB	24G1	B	B	B	H5	B	N1	A	B
		D064/25	H5N1	BBB5B1AB	24G1	B	B	B	H5	B	N1	A	B
		C071/25	H5N1	BBB5B1AB	24G1	B	B	B	H5	B	N1	A	B
		C090/25	H5N1	BBB5B1AB	24G1	B	B	B	H5	B	N1	A	B
		C095/25	H5N1	BBB5B1AB	24G1	B	B	B	H5	B	N1	A	B
		D135/25	H5N1	BBB5B1AB	24G1	B	B	B	H5	B	N1	A	B
		C161/25	H5N1	BBB5B1AB	24G1	B	B	B	H5	B	N1	A	B
		C194/25	H5N1	BBB5B1AB	24G1	B	B	B	H5	B	N1	A	B
		C195/25	H5N1	BBB5B1AB	24G1	B	B	B	H5	B	N1	A	B
		C198/25	H5N1	BBB5B1AB	24G1	B	B	B	H5	B	N1	A	B
		C199/25	H5N1	BBB5B1AB	24G1	B	B	B	H5	B	N1	A	B
		C200/25	H5N1	BBB5B1AB	24G1	B	B	B	H5	B	N1	A	B
		C201/25	H5N1	BBB5B1AB	24G1	B	B	B	H5	B	N1	A	B
		C214/25	H5N1	BBB5B1AB	24G1	B	B	B	H5	B	N1	A	B
		C215/25	H5N1	BBB5B1AB	24G1	B	B	B	H5	B	N1	A	B
		D216/25	H5N1	BBB5B1AB	24G1	B	B	B	H5	B	N1	A	B
		C268/25	H5N1	BBB5B1AB	24G1	B	B	B	H5	B	N1	A	B

In the 24/25 season, five distinct genotypes of H5N1 viruses (24G0–G4) were identified (Figure 2B and Table 1). Multiple genotypes were observed in Korea at the beginning of the season (October–December 2024). However, after December, the 24G1 became the dominant genotype circulating in poultry farms and wild birds. These results are consistent with the outbreak patterns observed in previous seasons, in which multiple strains appeared during the first 1–2 months, but a single genotype, likely the most suited to the region, eventually became dominant and persisted until the end of the season [18]. Interestingly, the same 24G1 genotype was identified in an H5N1 HPAIV from Mongolia in 2024 (An et al., unpublished data) and in an H5N1 HPAIV isolated in Japan (A/duck/Tottori/NK37‐40/2024; GISAID no.: EPL_ISL_19741594), indicating the dissemination of the 24G1 genotype across East Asia during the 2024–2025 season. (Figure 3B). In addition, the H5N1 genotype 22G0, which was detected in Korea in the 22/23 season [18], has been circulating worldwide, including in Europe, America, and Asia, since 2020 (classified as the A3 genotype in North America [GenoFLU] and the C genotype in Europe [Genin2]). This genotype was identified in South Korea during two consecutive winter seasons (23G0(23/24) and 24G0(24/25)), as well as in H5N1 HPAIVs isolated in Japan (Figure 3B, Figure S7) [31, 35]. These data suggest that the global circulation of H5N1 HPAIVs since 2021 continued to affect Korea and East Asia up to the 24/25 season.

### 3.4. H5N3 HPAI Virus in the 2024–2025 Winter Season

During the 24/25 winter season, a novel H5N3 HPAIV (A/northern pintail/Korea/WF369‐1/2024) was first identified in northern pintail on October 2, 2024. Phylogenetic analysis of the HA gene revealed that H5N3 HPAIV was closely related to the G2d group of clade 2.3.4.4b H5N1 HPAIV isolates (Figure 1) and that the N3 genes clustered with Eurasian low pathogenicity avian influenza (LPAI) viruses, including those from Korea and Japan (Supporting Information 1: Figure S3). When each gene segment of the H5N3 HPAIV was queried in the GISAID database, the closest matches were mostly clade 2.3.4.4b H5 HPAIVs (Table 2), except for the NA gene, which originated from Eurasian LPAIVs. Moreover, genome constellation analysis revealed that H5N3 HPAIV grouped as a 24G0 genotype with the N3 gene (Table 1) and showed low genetic similarity with reported H5N3 HPAIVs in Europe and Taiwan (Supporting Information 1: Table S2) [36–39]. These results suggest that the H5N3 HPAIV isolated in South Korea is genetically distinct from previous H5N3 strains. In addition, the virus may represent a reassortant between clade 2.3.4.4b H5 HPAIVs and the N3 genes from Eurasian LPAIVs (Tables 1 and 2). Notably, the H5N3 HPAIV was isolated from northern pintail, a typical winter‐migratory wild bird in South Korea, which is known to breed in the Siberia, Alaska, and Arctic regions and migrates to spend the winter in Korea, Japan, China, and South Asia. We assumed that reassortment may have occurred at breeding sites involving clade 2.3.4.4b H5 HPAIVs and Eurasian LPAIVs, after which the virus was subsequently introduced via migratory wild birds.

**Table 2:** Nucleotide sequence identities between novel H5N3 HPAIV(WF369‐1/2024) genes and closest viruses in the GISAID database.

Gene	Virus	GISAID accession no.	% identity	Sample date	LPAIV/ HPAIV H5 clade
PB2	A/large‐billed crow/Iwate/0303G012/2024(H5N1)	EPI_ISL_19139299	99.47	2024 Mar 13	2.3.4.4b
PB1	A/duck/Korea/D502/2023(H5N1)	EPI_ISL_18819797	99.74	2023 Dec 20	2.3.4.4b
PA	A/Eurasian jay/Hokkaido/20240311001/2024(H5N1)	EPI_ISL_19139301	99.74	2024 Mar 11	2.3.4.4b
HA	A/duck/Tottori/NK40/2023(H5N1)	EPI_ISL_18935784	99.82	2023 Nov 9	2.3.4.4b
NP	A/large‐billed crow/Iwate/0303G012/2024(H5N1)	EPI_ISL_19139299	99.73	2024 Mar 13	2.3.4.4b
NA	A/duck/Bangladesh/19‐D‐2278/2023(H2N3)	EPI_ISL_19187912	98.79	2023 Nov 22	LPAIV
M	A/Eurasian wigeon/Korea/23WS022‐22/2023(H5N1)	EPI_ISL_18717640	99.90	2023 Nov 27	2.3.4.4b
NS	A/Eurasian wigeon/Korea/23WS022‐22/2023(H5N1)	EPI_ISL_18717640	99.64	2023 Nov 27	2.3.4.4b

Abbreviations: HA, hemagglutinin; M, matrix; NA, neuraminidase; NP, nucleoprotein; NS, nonstructural; PA, polymerase acidic; PB1, polymerase basic 1; PB2, polymerase basic 2.

## 4. Conclusion

In this study, we present the emergence and genetic characteristics of H5N1, H5N6, and H5N3 HPAIVs isolated in South Korea during the 23/24 and 24/25 winter seasons. H5N6 and H5N1 HPAIVs cocirculated in the 23/24 season and were isolated from poultry and wild birds, whereas H5N3 and H5N1 HPAIVs were detected in the 24/25 season, also from poultry and wild birds. Notably, a novel H5N3 HPAIV isolated from wild bird was identified as a reassortant virus distinct from previously reported H5N3 HPAIVs but genetically similar to clade 2.3.4.4b H5 HPAI viruses except NA gene. In both seasons, the HA genes were closely related to H5 HPAIVs isolated from Eurasia, and multiple genotypes were detected. We also identified the same genotypes in clade 2.3.4.4b H5Nx HPAIVs isolated from neighboring East Asian countries around the same time. These findings suggest that diverse reassorted clade 2.3.4.4b H5 HPAIVs may be introduced by migratory wild birds. The virus is best adapted to wild birds, most compatible with local environmental conditions, and may become the dominant genotype that causes outbreaks in domestic poultry populations. However, further studies may be needed to analyze the dynamic of HPAI virus reassortment and evolution. Taken together, our results emphasize that understanding the genetic characteristics of clade 2.3.4.4b H5Nx HPAIVs is crucial for inferring routes of viral introduction and establishing effective preventive measures in the field.

## Funding

This work was supported by the Animal and Plant Quarantine Agency (APQA) of the Ministry of Agriculture, Food, and Rural Affairs of the Republic of Korea (Grant B‐1543418‐2024‐26‐0102).

## Conflicts of Interest

The authors declare no conflicts of interest.

## Supporting Information

Additional supporting information can be found online in the Supporting Information section.

## Supporting information


**Supporting Information 1** Figure S1. Weekly prevalence of H5N1, H5N6, and H5N3 HPAIV in wild birds (A) and poultry (B) during the 23/24 and 24/25 seasons. Figure S2. Regional prevalence of H5N1, H5N6, and H5N3 HPAIV in wild birds and poultry during the 23/24 (A) and 24/25 (B) seasons. Poultry birds (red), wild birds (green), and both species (yellow) are shown. Figure S3. Regional prevalence of genotypes of H5N1, H5N6, and H5N3 HPAIV in poultry and wild birds during the 23/24 (A) and 24/25 (B) seasons. Poultry (circle) and wild birds (triangle) were shown. Figure S4. tMRCA estimation of MCC tree of HA gene of Korean H5Nx HPAI virus isolates from 23/24 and 24/25 seasons. Blue: H5Nx HPAIVs isolated in 23/24 season; red: H5Nx HPAIVs isolated in 24/25 season. Figure S5. Maximum‐likelihood phylogenetic tree of the NA gene of Korean H5Nx HPAI isolates from the 23/24 and 24/25 seasons. N1 (A), N6 (B), and N3 (C) trees. Blue: H5Nx HPAIVs isolated in 23/24 season; red: H5Nx HPAIVs isolated in 24/25 season. Figure S6. Maximum‐likelihood phylogenetic tree of internal gene segments for the gene constellation. Figure S7. Schematic diagram of genomic constellation of 23/24 and 24/25 H5N1, H5N6, and H5N3 HPAI viruses. Table S1. Clade 2.3.4.4 b H5N6, H5N1, and H5N3 HPAIVs isolated in this study. Table S2. Nucleotide sequence identities (%) between genes of a novel H5N3 HPAIV (WF369‐1/2024) genes and previously reported H5N3 HPAIV viruses.

**Supporting Information 2** Table S3. Reference virus sequence information used for the HA and NA (N1, N6, and N3) phylogenetic tree analysis.

## Data Availability

All sequence data generated in this study were deposited and are available at the GISAID database (https://platform.gisaid.org).
