# Supplementary material for: Emergence and Genetic Characteristics of H5N1, H5N6, and H5N3 Clade 2.3.4.4b High Pathogenicity Avian Influenza Viruses in South Korea During the 2023–2024 and 2024–2025 Winter Seasons
Source: Transbound Emerg Dis. 2026 Feb 24;2026:8053623. doi: 10.1155/tbed/8053623 (PMC12931157; doi:10.1155/tbed/8053623)
Supplement: Supplementary file 1 — Supporting Information 1 Figure S1. Weekly prevalence of H5N1, H5N6, and H5N3 HPAIV in wild birds (A) and poultry (B) during the 23/24 and 24/25 seasons. Figure S2. Regional prevalence of H5N1, H5N6, and H5N3 HPAIV in wild birds and poultry during the 23/24 (A) and 24/25 (B) seasons. Poultry birds (red), wild birds (green), and both species (yellow) are shown. Figure S3. Regional prevalence of genotypes of H5N1, H5N6, and H5N3 HPAIV in poultry and wild birds during the 23/24 (A) and 24/25 (B) seasons. Poultry (circle) and wild birds (triangle) were shown. Figure S4. tMRCA estimation of MCC tree of HA gene of Korean H5Nx HPAI virus isolates from 23/24 and 24/25 seasons. Blue: H5Nx HPAIVs isolated in 23/24 season; red: H5Nx HPAIVs isolated in 24/25 season. Figure S5. Maximum‐likelihood phylogenetic tree of the NA gene of Korean H5Nx HPAI isolates from the 23/24 and 24/25 seasons. N1 (A), N6 (B), and N3 (C) trees. Blue: H5Nx HPAIVs isolated in 23/24 season; red: H5Nx HPAIVs isolated in 24/25 season. Figure S6. Maximum‐likelihood phylogenetic tree of internal gene segments for the gene constellation. Figure S7. Schematic diagram of genomic constellation of 23/24 and 24/25 H5N1, H5N6, and H5N3 HPAI viruses. Table S1. Clade 2.3.4.4 b H5N6, H5N1, and H5N3 HPAIVs isolated in this study. Table S2. Nucleotide sequence identities (%) between genes of a novel H5N3 HPAIV (WF369‐1/2024) genes and previously reported H5N3 HPAIV viruses. [file TBED-2026-8053623-s002.docx]

**Supplementary Data**

1. Wild birds
2. Poultry

**Figure S1.** Weekly prevalence of H5N1, H5N6, and H5N3 HPAIV in wild birds(A) and poultry(B) during the 23/24 and 24/25 seasons.


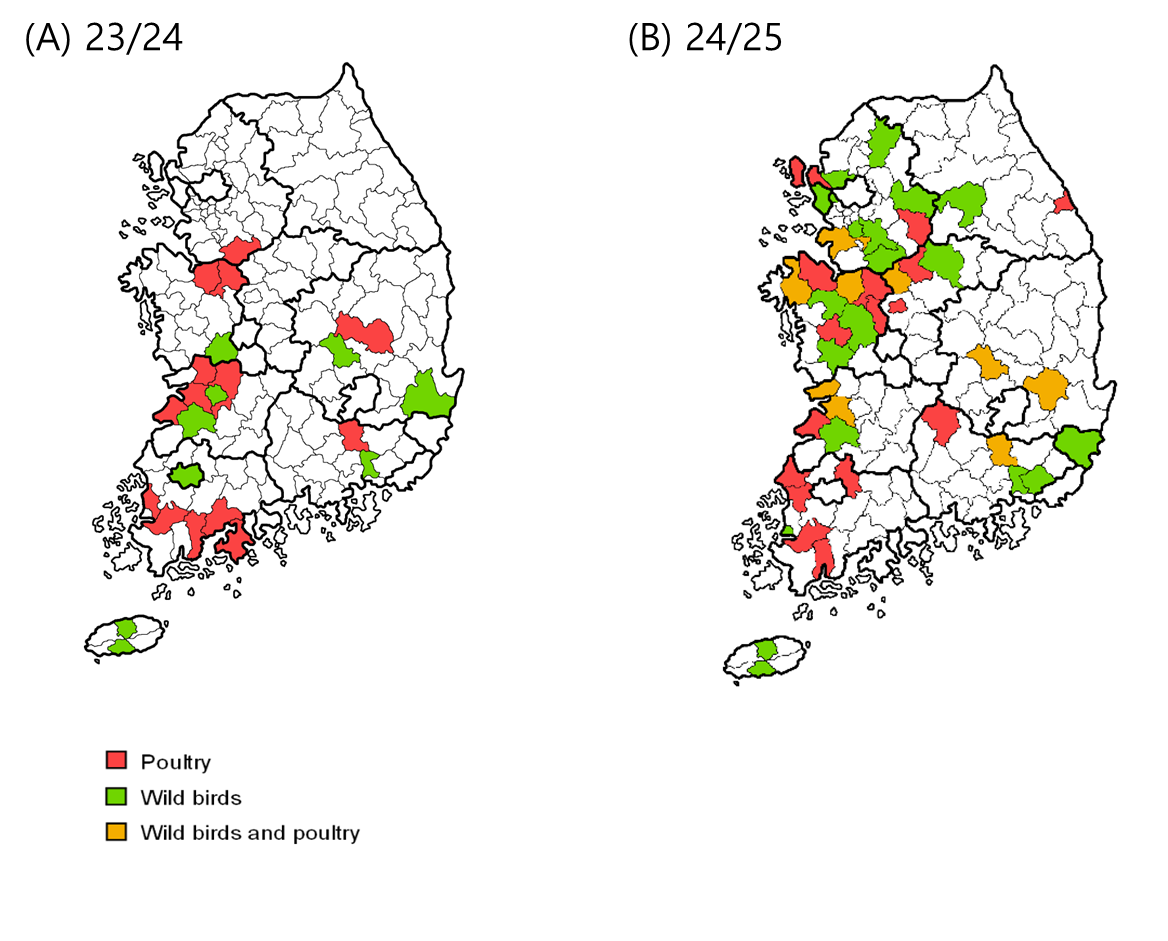


**Figure S2.** Regional prevalence of H5N1, H5N6, and H5N3 HPAIV in wild bird and poultry during the 23/24 (A) and 24/25 (B) seasons. poultry (red), wild birds (green) or both (yellow) were shown.


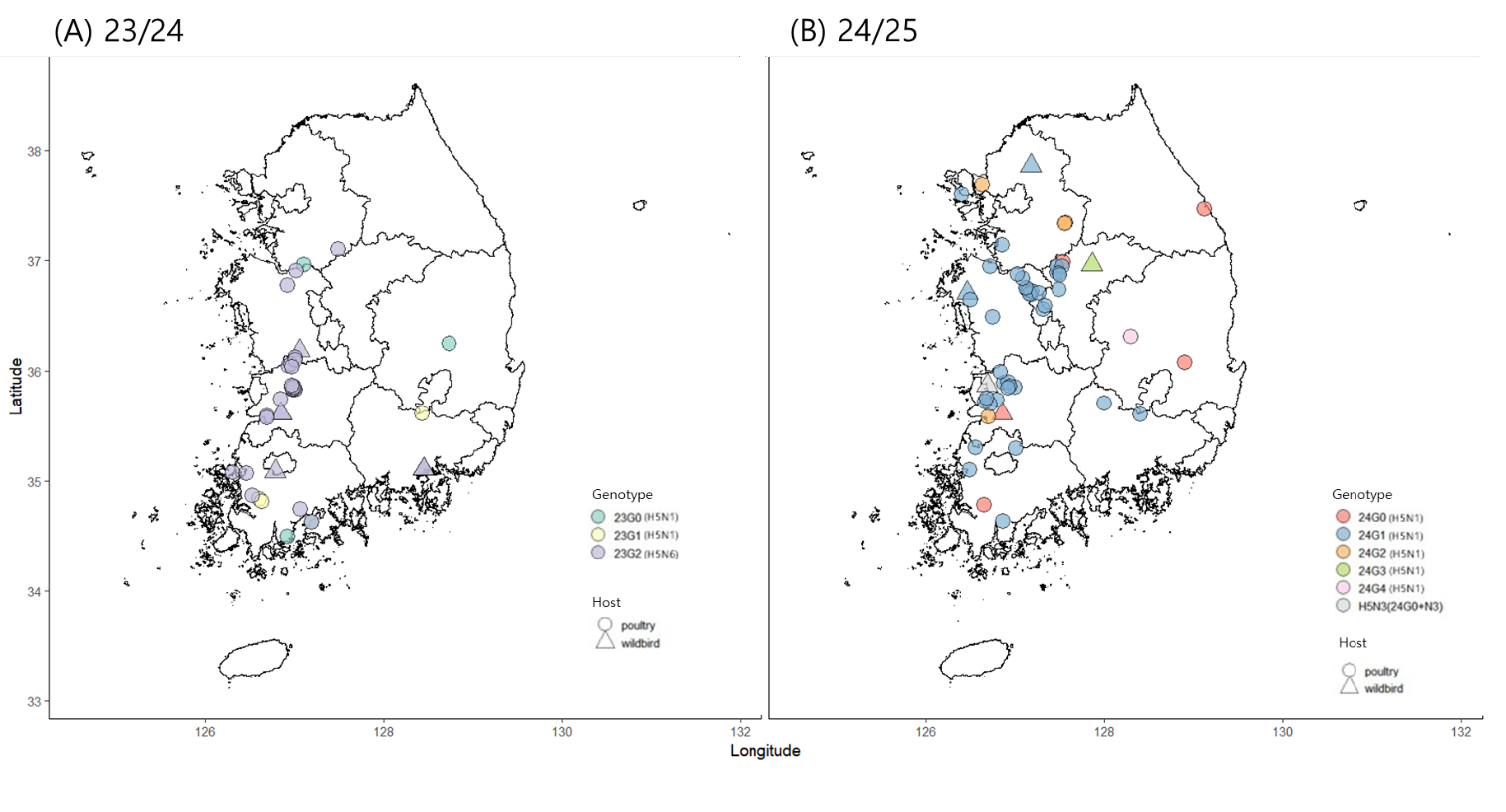


**Figure S3.** Regional prevalence of genotypes of H5N1, H5N6, and H5N3 HPAIV in poultry and wild birds during the 23/24 (A) and 24/25 (B) seasons. Poultry (circle), wild birds (triangle) were shown.


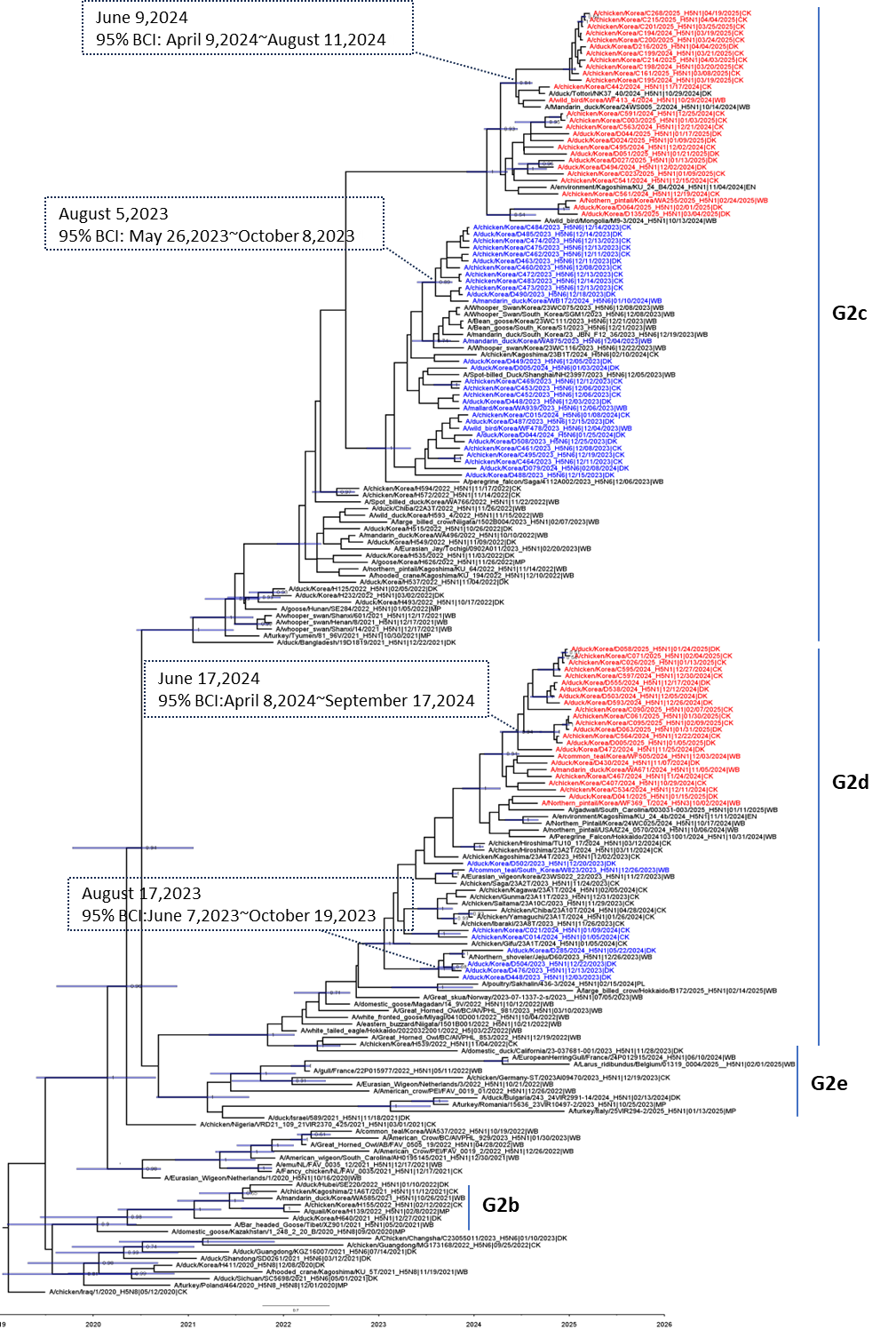


**Figure S4**. tMRCA estimation of MCC tree of HA gene of Korean H5Nx HPAI viruses isolates from 23/24 and 24/25 seasons. Blue: H5Nx HPAIVs isolated in 23/24 season; Red: H5Nx HPAIVs isolated in 24/25 season


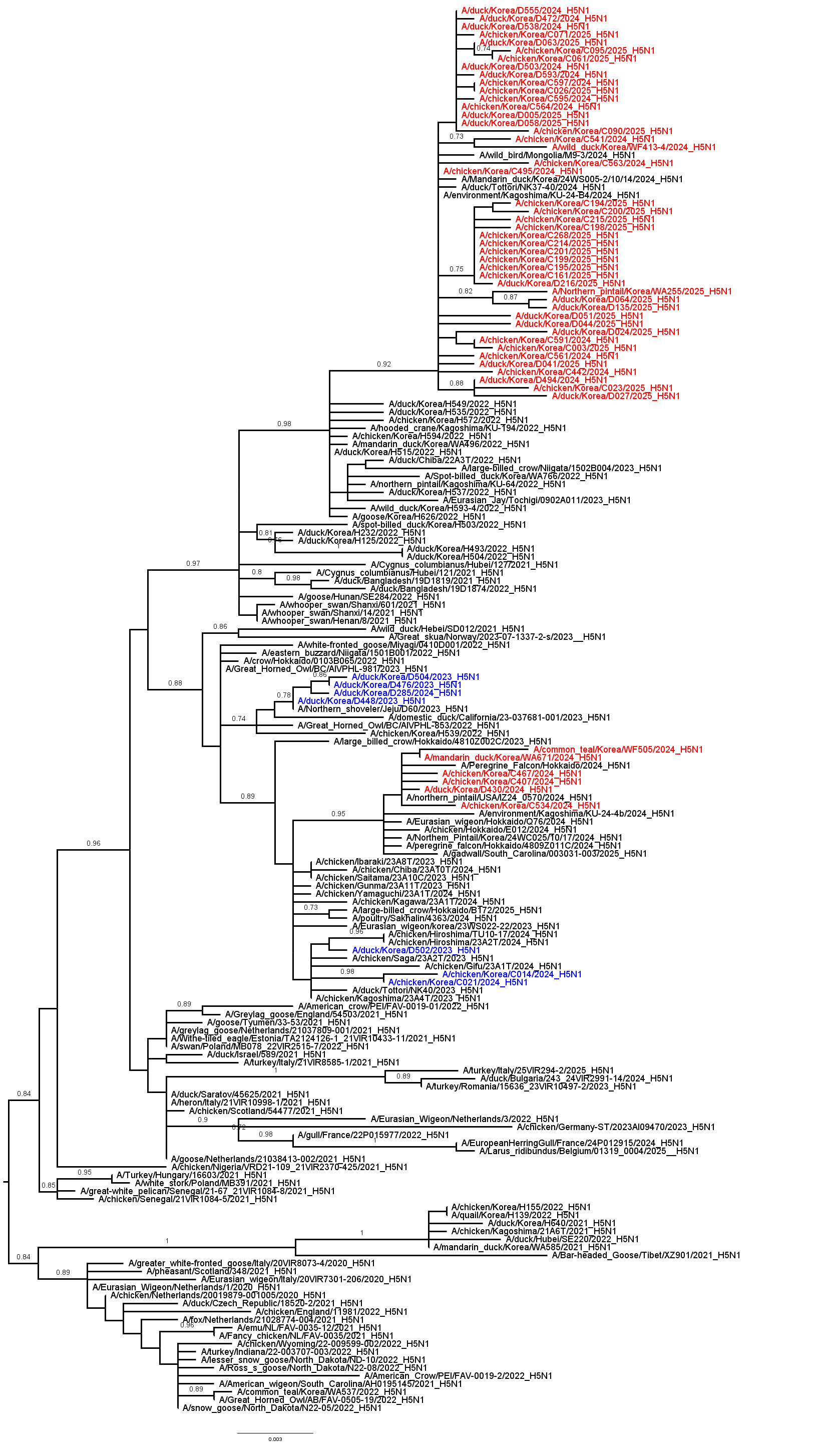
(A) N1


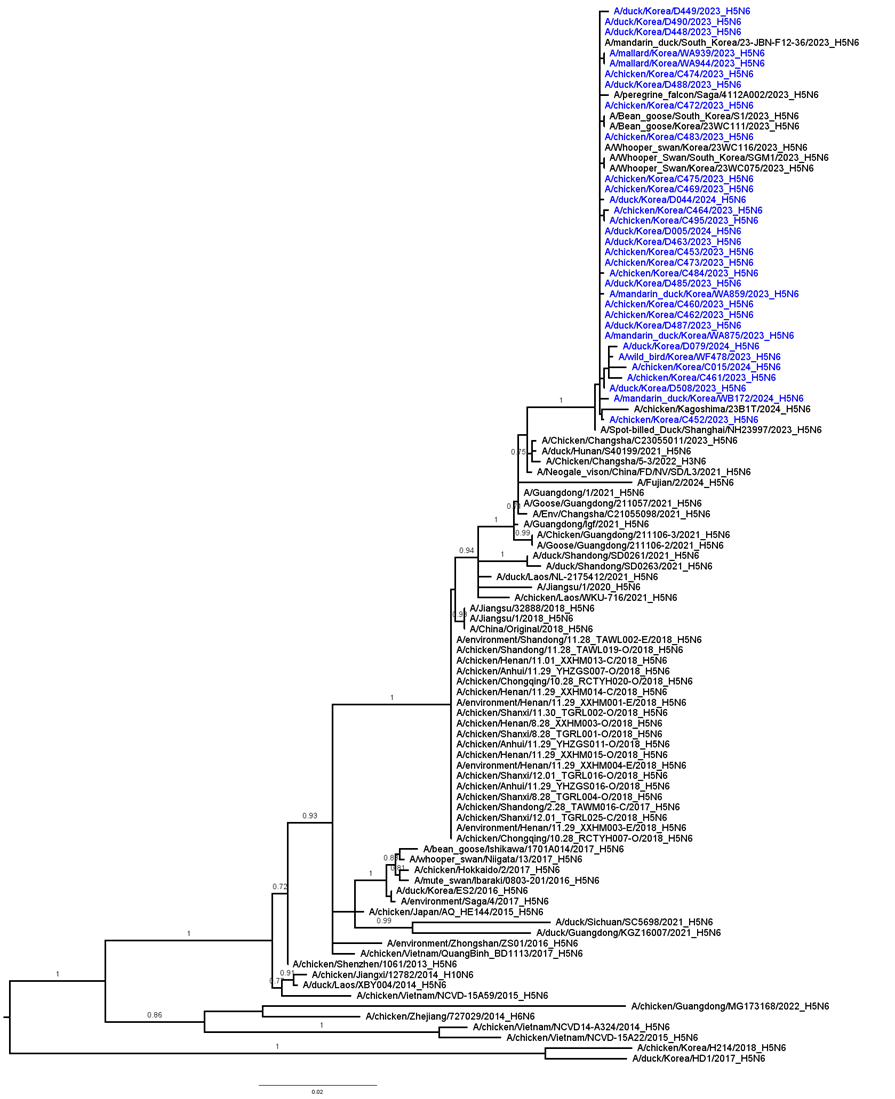
(B) N6

(C) N3

**
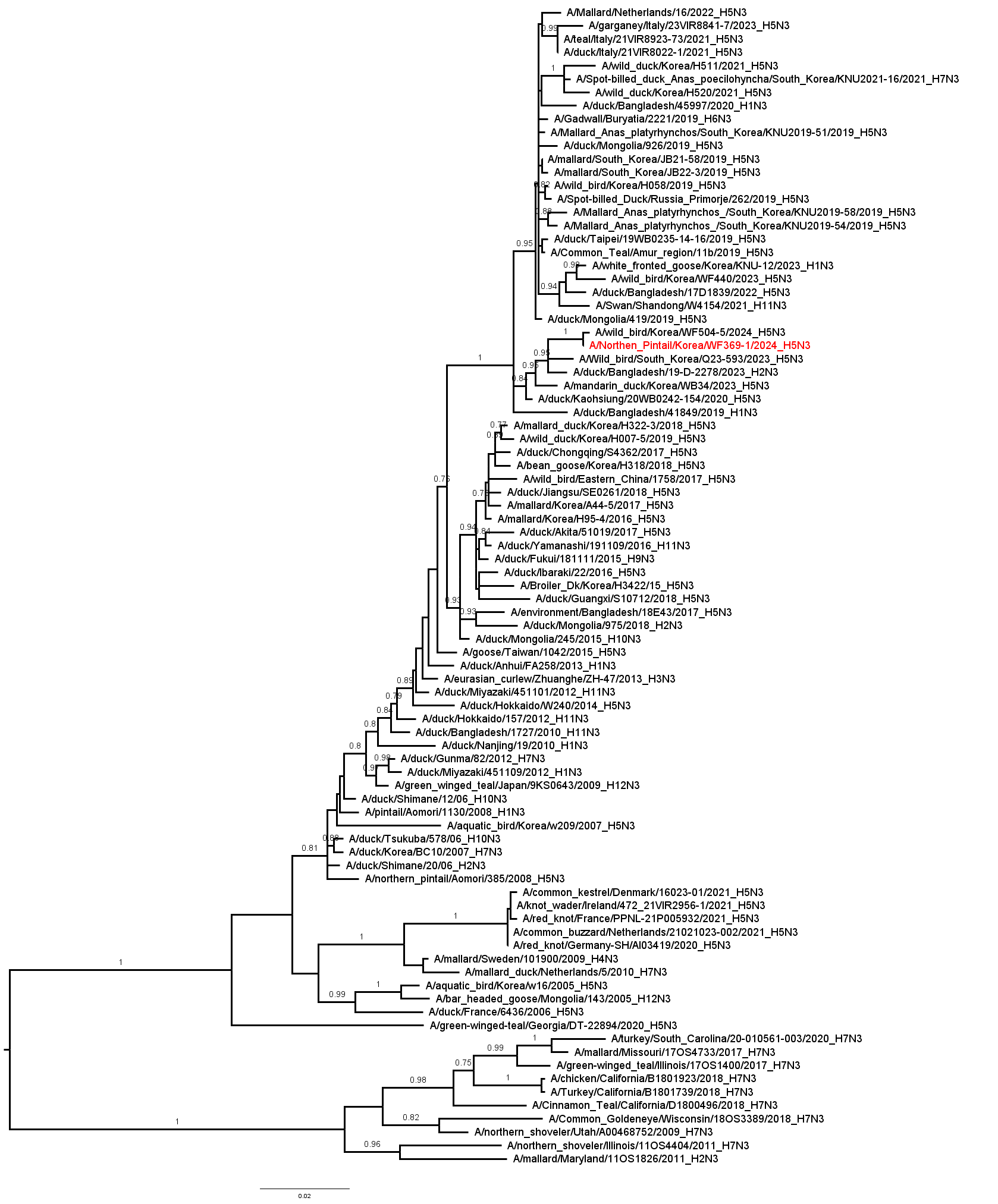
**

**Figure S5.** Maximum-likelihood phylogenetic tree of the NA gene of Korean H5Nx HPAI viruses isolates from 23/24 and 24/25 seasons. N1(A), N6(B) and N3(C) tree were shown. Blue: H5Nx HPAIVs isolated in 23/24 season; Red: H5Nx HPAIVs isolated in 24/25 season


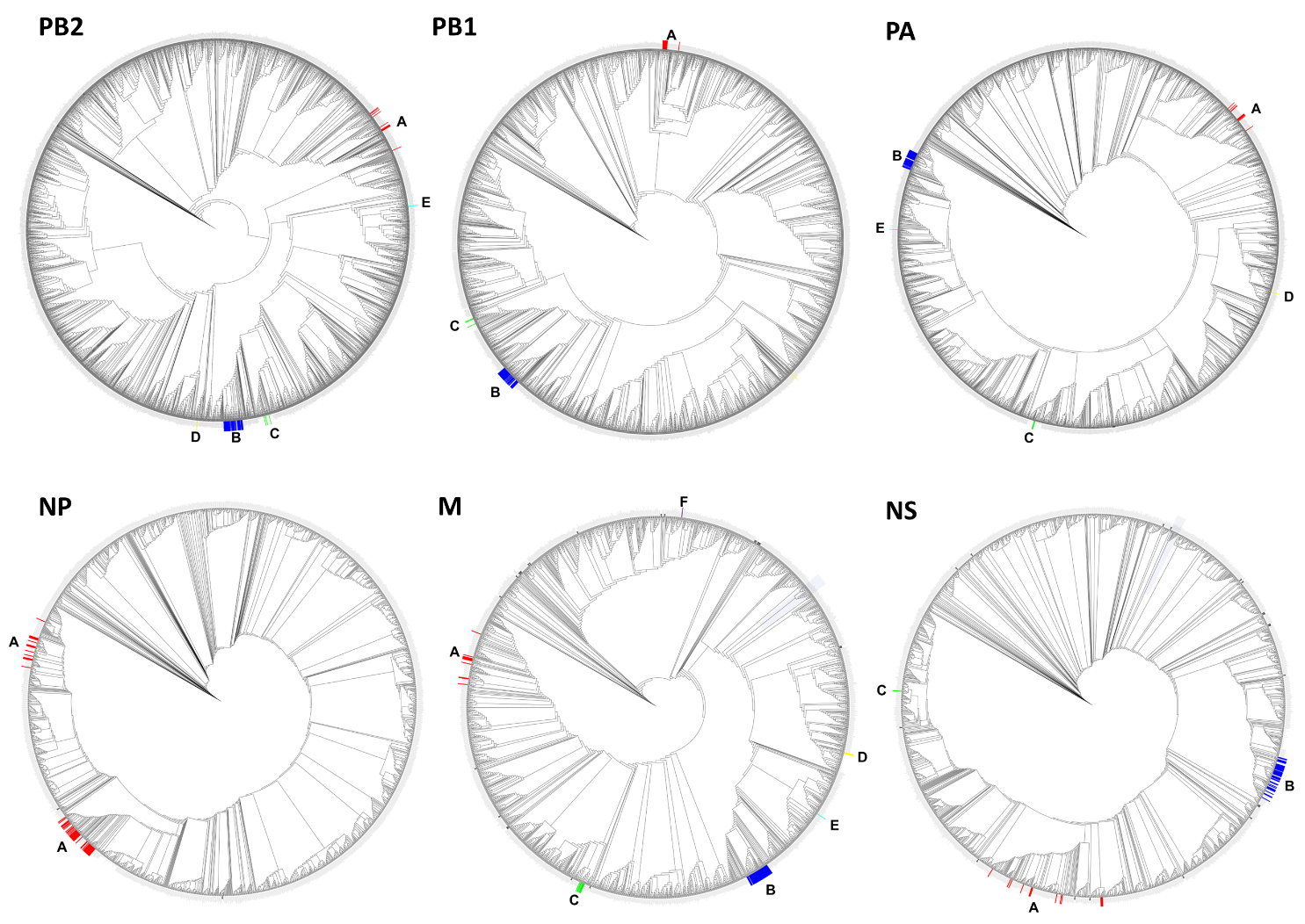


**Figure S6**. Maximum-likelihood phylogenetic tree of internal gene segments for gene constellation


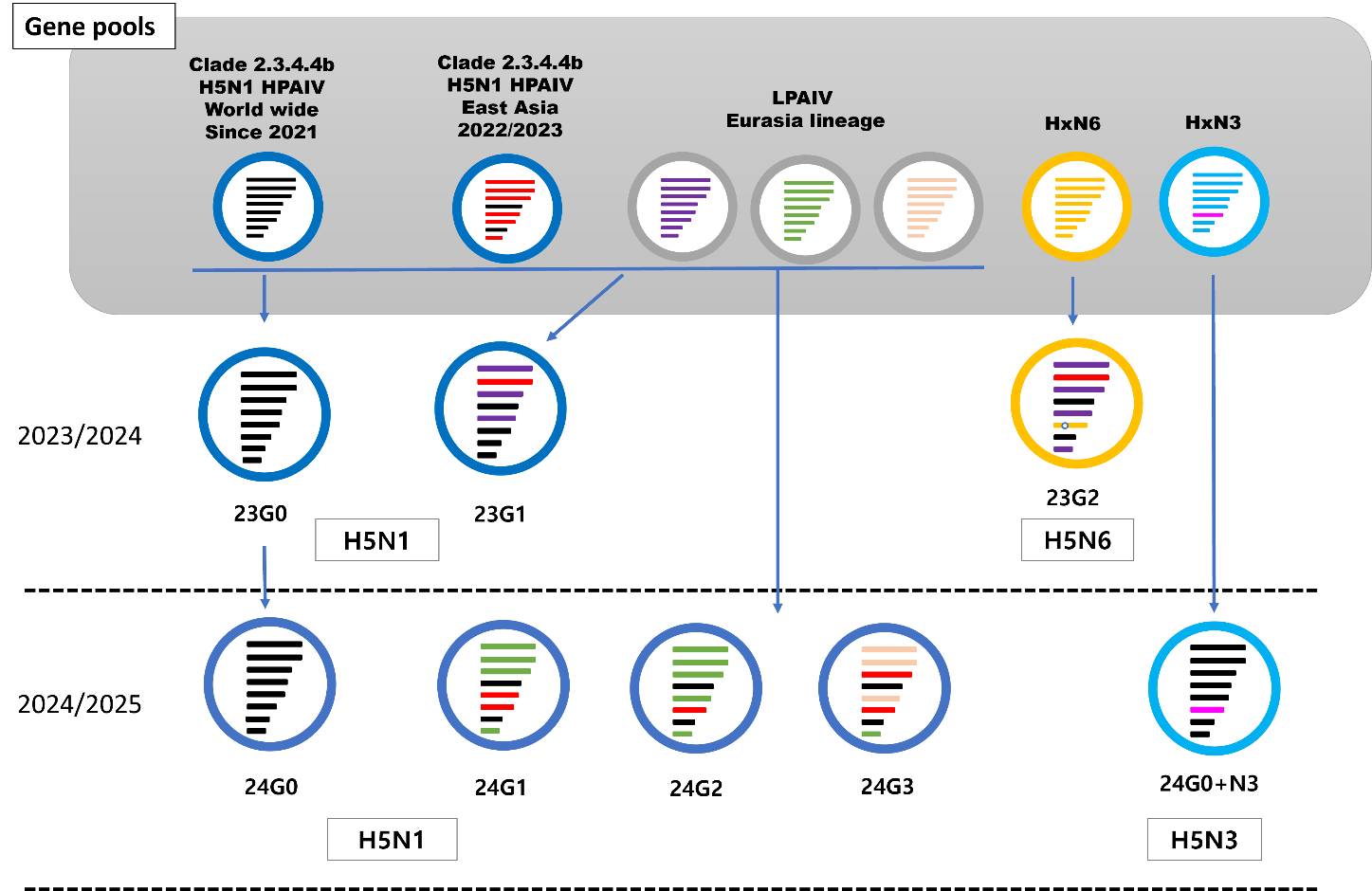


**Figure S7**. Schematic diagram of genomic constellation of 23/24 and 24/25 H5N1, H5N6 and H5N3 HPAI viruses

Table S1. Clade 2.3.4.4b H5N6, H5N1 and H5N3 HPAIVs isolated in this study

| Season | Host |  | Virus name | Collection date | Region | Genotype | Sample type | latitude | longitude | Cleavage site | GISIAD Isolate no |
| --- | --- | --- | --- | --- | --- | --- | --- | --- | --- | --- | --- |
| 23/24 | Poultry | Duck | A/duck/Korea/D448_N1/2023(H5N1) | 2023-12-03 | JN | 23G0 | Swab/Organ | 34°37'31" | 127°11'27" | PLREKRRKR*GLF | EPI_ISL_19884233 |
|  |  | Duck | A/duck/Korea/D476/2023(H5N1) | 2023-12-13 | JN | 23G1 | Swab/Organ | 34°50'17" | 126°36'9" | PLREKRRKR*GLF | EPI_ISL_19884237 |
|  |  | Duck | A/duck/Korea/D502/2023(H5N1) | 2023-12-20 | JN | 23G0 | Swab | 34°29'42" | 126°55'14" | PLREKRRKR*GLF | EPI_ISL_19884242 |
|  |  | Duck | A/duck/Korea/D504/2023(H5N1) | 2023-12-22 | JN | 23G1 | Swab/Organ | 34°48'42" | 126°38'12" | PLREKRRKR*GLF | EPI_ISL_19884243 |
|  |  | Chicken | A/chicken/Korea/C014/2024(H5N1) | 2024-01-05 | CN | 23G0 | Organ | 36°57'45" | 127°6'36" | PLREKRRKR*GLF | EPI_ISL_19884212 |
|  |  | Chicken | A/chicken/Korea/C021/2024(H5N1) | 2024-01-09 | GB | 23G0 | Organ | 36°14'55" | 128°43'59" | PLREKRRKR*GLF | EPI_ISL_19884214 |
|  |  | Duck | A/duck/Korea/D285/2024(H5N1) | 2024-05-22 | GN | 23G1 | Swab/Feces | 35°36'44" | 128°25'50" | PLREKRRKR*GLF | EPI_ISL_19884232 |
|  | Wild bird | Wild bird | A/wild bird/Korea/WF478/2023(H5N6) | 2023-12-04 | CN | 23G2 | Swab | 36°11'19" | 127°3'29" | PLREKRRKR*GLF | EPI_ISL_19884250 |
|  |  | Mandarin duck | A/mandarin duck/Korea/WA859/2023(H5N6) | 2023-12-04 | JB | 23G2 | Swab | 35°36'33" | 126°50'60" | PLREKRRKR*GLF | EPI_ISL_19884247 |
|  |  | Mandarin duck | A/mandarin duck/Korea/WA875/2023(H5N6) | 2023-12-04 | JB | 23G2 | Swab | 35°36'33" | 126°50'60" | PLREKRRKR*GLF | EPI_ISL_19884248 |
|  |  | Mallard | A/mallard/Korea/WA939/2023(H5N6) | 2023-12-06 | GN | 23G2 | Swab | 35°6'21" | 128°26'41" | PLREKRRKR*GLF | EPI_ISL_19884245 |
|  |  | Mallard | A/mallard/Korea/WA944/2023(H5N6) | 2023-12-06 | GN | 23G2 | Swab | 35°6'21" | 128°26'41" | PLREKRRKR*GLF | EPI_ISL_19884246 |
|  |  | Mandarin duck | A/mandarin duck/Korea/WB172/2024(H5N6) | 2024-01-10 | JN | 23G2 | Swab | 35°5'12" | 126°47'13" | PLREKRRKR*GLF | EPI_ISL_19884249 |
|  | Poultry | Duck | A/duck/Korea/D448_N6/2023(H5N6) | 2023-12-03 | JN | 23G2 | Swab/Organ | 34°37'31" | 127°11'27" | PLREKRRKR*GLF | EPI_ISL_19884234 |
|  |  | Duck | A/duck/Korea/D449/2023(H5N6) | 2023-12-05 | JN | 23G2 | Swab | 34°52'11" | 126°32'2" | PLREKRRKR*GLF | EPI_ISL_19884235 |
|  |  | Chicken | A/chicken/Korea/C452/2023(H5N6) | 2023-12-06 | JB | 23G2 | Organ | 36°7'31" | 127°0'19" | PLREKRRKR*GLF | EPI_ISL_19884215 |
|  |  | Chicken | A/chicken/Korea/C453/2023(H5N6) | 2023-12-06 | JB | 23G2 | Organ | 36°2'48" | 126°55'59" | PLREKRRKR*GLF | EPI_ISL_19884216 |
|  |  | Chicken | A/chicken/Korea/C460/2023(H5N6) | 2023-12-08 | JB | 23G2 | Swab/Organ | 35°49'52" | 126°59'0" | PLREKRRKR*GLF | EPI_ISL_19884217 |
|  |  | Chicken | A/chicken/Korea/C461/2023(H5N6) | 2023-12-08 | CN | 23G2 | Organ | 36°46'30" | 126°55'14" | PLREKRRKR*GLF | EPI_ISL_19884218 |
|  |  | Chicken | A/chicken/Korea/C462/2023(H5N6) | 2023-12-11 | JB | 23G2 | Swab/Organ/Feces | 35°51'42" | 126°59'42" | PLREKRRKR*GLF | EPI_ISL_19884219 |
|  |  | Duck | A/duck/Korea/D463/2023(H5N6) | 2023-12-11 | JB | 23G2 | Swab/Organ | 35°49'32" | 127°0'36" | PLREKRRKR*GLF | EPI_ISL_19884236 |
|  |  | Chicken | A/chicken/Korea/C464/2023(H5N6) | 2023-12-11 | JB | 23G2 | Swab/Organ | 36°3'44" | 126°57'44" | PLREKRRKR*GLF | EPI_ISL_19884220 |
|  |  | Chicken | A/chicken/Korea/C469/2023(H5N6) | 2023-12-12 | JB | 23G2 | Organ/Feces | 36°6'11" | 127°0'25" | PLREKRRKR*GLF | EPI_ISL_19884221 |
|  |  | Chicken | A/chicken/Korea/C472/2023(H5N6) | 2023-12-13 | JB | 23G2 | Organ | 35°50'5" | 127°0'2" | PLREKRRKR*GLF | EPI_ISL_19884222 |
|  |  | Chicken | A/chicken/Korea/C473/2023(H5N6) | 2023-12-13 | JB | 23G2 | Organ | 35°50'21" | 126°58'48" | PLREKRRKR*GLF | EPI_ISL_19884223 |
|  |  | Chicken | A/chicken/Korea/C474/2023(H5N6) | 2023-12-13 | JB | 23G2 | Organ | 35°50'23" | 126°58'50" | PLREKRRKR*GLF | EPI_ISL_19884224 |
|  |  | Chicken | A/chicken/Korea/C475/2023(H5N6) | 2023-12-13 | JB | 23G2 | Organ | 35°50'20" | 126°58'49" | PLREKRRKR*GLF | EPI_ISL_19884225 |
|  |  | Chicken | A/chicken/Korea/C483/2023(H5N6) | 2023-12-14 | JB | 23G2 | Organ | 35°51'8" | 126°58'27" | PLREKRRKR*GLF | EPI_ISL_19884226 |
|  |  | Chicken | A/chicken/Korea/C484/2023(H5N6) | 2023-12-14 | JB | 23G2 | Organ | 35°51'1" | 126°59'20" | PLREKRRKR*GLF | EPI_ISL_19884227 |
|  |  | Duck | A/duck/Korea/D485/2023(H5N6) | 2023-12-14 | JB | 23G2 | Organ | 35°51'56" | 126°58'13" | PLREKRRKR*GLF | EPI_ISL_19884238 |
|  |  | Duck | A/duck/Korea/D487/2023(H5N6) | 2023-12-15 | JB | 23G2 | Organ | 35°35'29" | 126°41'30" | PLREKRRKR*GLF | EPI_ISL_19884239 |
|  |  | Duck | A/duck/Korea/D488/2023(H5N6) | 2023-12-15 | JB | 23G2 | Organ | 35°34'36" | 126°41'35" | PLREKRRKR*GLF | EPI_ISL_19884240 |
|  |  | Duck | A/duck/Korea/D490/2023(H5N6) | 2023-12-18 | JB | 23G2 | Organ | 35°44'25" | 126°50'39" | PLREKRRKR*GLF | EPI_ISL_19884241 |
|  |  | Chicken | A/chicken/Korea/C495/2023(H5N6) | 2023-12-19 | JB | 23G2 | Organ | 36°2'33" | 126°58'14" | PLREKRRKR*GLF | EPI_ISL_19884228 |
|  |  | Duck | A/duck/Korea/D508/2023(H5N6) | 2023-12-25 | JN | 23G2 | Organ | 34°44'36" | 127°4'19" | PLREKRRKR*GLF | EPI_ISL_19884244 |
|  |  | Duck | A/duck/Korea/D005/2024(H5N6) | 2024-01-03 | JN | 23G2 | Swab/Organ | 35°4'44" | 126°18'26" | PLREKRRKR*GLF | EPI_ISL_19884229 |
|  |  | Chicken | A/chicken/Korea/C015/2024(H5N6) | 2024-01-08 | GG | 23G2 | Organ | 37°6'2" | 127°29'20" | PLREKRRKR*GLF | EPI_ISL_19884213 |
|  |  | Duck | A/duck/Korea/D044/2024(H5N6) | 2024-01-25 | JN | 23G2 | Organ | 35°4'18" | 126°27'36" | PLREKRRKR*GLF | EPI_ISL_19884230 |
|  |  | Duck | A/duck/Korea/D079/2024(H5N6) | 2024-02-08 | CN | 23G2 | Swab | 36°54'21" | 127°0'58" | PLREKRRKR*GLF | EPI_ISL_19884231 |
| 24/25 | Wild bird | Northen pintail | A/Northern pintail/Korea/WF369-1/2024(H5N3) | 2024-10-02 | JB | H5N3(24G0+N3) | Feces | 35°52'18" | 126°41'9" | PLREKRRKR*GLF | EPI_ISL_19884294 |
|  |  | Wild duck | A/wild bird/Korea/WF413/2024(H5N1) | 2024-10-29 | GG | 24G1 | Organ | 37°51'40" | 127°10'29" | PLREKRRKR*GLF | EPI_ISL_19825819 |
|  |  | Mandarin duck | A/mandarin duck/Korea/WA671/2024(H5N1) | 2024-11-05 | JB | 24G0 | Swab | 35°36'34" | 126°51'0" | PLREKRRKR*GLF | EPI_ISL_19884292 |
|  |  | Common teal | A/common teal/Korea/WF505/2024(H5N1) | 2024-12-03 | CB | 24G3 | Feces | 36°58'3" | 127°51'54" | PLREKRRKR*GLF | EPI_ISL_19884274 |
|  |  | Northen pintail | A/Northern pintail/Korea/WA255/2025(H5N1) | 2025-02-24 | CN | 24G1 | Swab | 36°42'37" | 126°27'42" | PLREKRRKR*GLF | EPI_ISL_19884293 |
|  | Poultry | Chicken | A/chicken/Korea/C407/2024(H5N1) | 2024-10-29 | GW | 24G0 | Organ | 37°28'12" | 129°7'36" | PLRGKRRKR*GLF | EPI_ISL_19884310 |
|  |  | Duck | A/duck/Korea/D430/2024(H5N1) | 2024-11-07 | CB | 24G0 | Organ | 36°59'7" | 127°32'11" | PLREKRRKR*GLF | EPI_ISL_19884285 |
|  |  | Chicken | A/chicken/Korea/C442/2024(H5N1) | 2024-11-17 | IC | 24G1 | Swab/Organ | 37°36'5" | 126°24'17" | PLREKRRKR*GLF | EPI_ISL_19825820 |
|  |  | Chicken | A/chicken/Korea/C467/2024(H5N1) | 2024-11-24 | JN | 24G0 | Organ | 34°46'42" | 126°38'60" | PLREKRRKR*GLF | EPI_ISL_19884311 |
|  |  | Duck | A/duck/Korea/D472/2024(H5N1) | 2024-11-25 | CN | 24G1 | Swab | 36°38'48" | 126°29'42" | PLREKRRKR*GLF | EPI_ISL_19884286 |
|  |  | Duck | A/duck/Korea/D494/2024(H5N1) | 2024-12-02 | JN | 24G1 | Organ | 34°38'3" | 126°51'28" | PLREKRRKR*GLF | EPI_ISL_19884287 |
|  |  | Chicken | A/chicken/Korea/C495/2024(H5N1) | 2024-12-02 | SJ | 24G1 | Organ | 36°33'28" | 127°18'37" | PLREKRRKR*GLF | EPI_ISL_19884312 |
|  |  | Duck | A/duck/Korea/D503/2024(H5N1) | 2024-12-05 | JB | 24G1 | Organ | 35°53'23" | 126°52'11" | PLREKRRKR*GLF | EPI_ISL_19884288 |
|  |  | Chicken | A/chicken/Korea/C534/2024(H5N1) | 2024-12-11 | GB | 24G0 | Organ | 36°4'34" | 128°53'57" | PLREKRRKR*GLF | EPI_ISL_19884313 |
|  |  | Duck | A/duck/Korea/D538/2024(H5N1) | 2024-12-12 | JB | 24G1 | Organ | 35°44'20" | 126°47'45" | PLREKRRKR*GLF | EPI_ISL_19884289 |
|  |  | Chicken | A/chicken/Korea/C541/2024(H5N1) | 2024-12-15 | CN | 24G1 | Organ | 36°29'21" | 126°44'43" | PLREKRRKR*GLF | EPI_ISL_19884314 |
|  |  | Duck | A/duck/Korea/D555/2024(H5N1) | 2024-12-17 | JB | 24G1 | Swab/Organ | 35°41'42" | 126°43'1" | PLREKRRKR*GLF | EPI_ISL_19884290 |
|  |  | Chicken | A/chicken/Korea/C561/2024(H5N1) | 2024-12-19 | GG | 24G2 | Swab | 37°41'8" | 126°37'33" | PLREKRRKR*GLF | EPI_ISL_19884268 |
|  |  | Chicken | A/chicken/Korea/C563/2024(H5N1) | 2024-12-21 | GG | 24G1 | Organ | 37°8'39" | 126°51'1" | PLREKRRKR*GLF | EPI_ISL_19884269 |
|  |  | Chicken | A/chicken/Korea/C564/2024(H5N1) | 2024-12-22 | JB | 24G1 | Swab/Organ | 35°51'23" | 126°59'38" | PLREKRRKR*GLF | EPI_ISL_19884270 |
|  |  | Chicken | A/chicken/Korea/C591/2024(H5N1) | 2024-12-25 | GG | 24G2 | Organ | 37°20'33" | 127°33'57" | PLREKRRKR*GLF | EPI_ISL_19884271 |
|  |  | Duck | A/duck/Korea/D593/2024(H5N1) | 2024-12-26 | JB | 24G1 | Organ | 35°43'13" | 126°39'41" | PLREKRRKR*GLF | EPI_ISL_19884291 |
|  |  | Chicken | A/chicken/Korea/C595/2024(H5N1) | 2024-12-27 | CB | 24G1 | Swab | 36°53'31" | 127°27'49" | PLREKRRKR*GLF | EPI_ISL_19884272 |
|  |  | Chicken | A/chicken/Korea/C597/2024(H5N1) | 2024-12-30 | CB | 24G1 | Organ | 36°57'3" | 127°27'42" | PLREKRRKR*GLF | EPI_ISL_19884273 |
|  |  | Chicken | A/chicken/Korea/C003/2025(H5N1) | 2025-01-03 | GG | 24G2 | Organ | 37°20'10" | 127°33'53" | PLREKRRKR*GLF | EPI_ISL_19884298 |
|  |  | Duck | A/duck/Korea/D005/2025(H5N1) | 2025-01-05 | JB | 24G1 | Organ | 35°53'46" | 126°55'7" | PLREKRRKR*GLF | EPI_ISL_19884275 |
|  |  | Chicken | A/chicken/Korea/C023/2025(H5N1) | 2025-01-09 | CN | 24G1 | Organ | 36°56'56" | 126°43'7" | PLREKRRKR*GLF | EPI_ISL_19884299 |
|  |  | Duck | A/duck/Korea/D024/2025(H5N1) | 2025-01-09 | JB | 24G2 | Organ | 35°34'51" | 126°41'59" | PLREKRRKR*GLF | EPI_ISL_19884276 |
|  |  | Chicken | A/chicken/Korea/C026/2025(H5N1) | 2025-01-13 | CB | 24G1 | Organ | 36°57'7" | 127°31'55" | PLREKRRKR*GLF | EPI_ISL_19884300 |
|  |  | Duck | A/duck/Korea/D027/2025(H5N1) | 2025-01-13 | GN | 24G1 | Swab/Feces | 35°36'24" | 128°24'23" | PLREKRRKR*GLF | EPI_ISL_19884277 |
|  |  | Duck | A/duck/Korea/D041/2025(H5N1) | 2025-01-15 | GN | 24G1 | Swab/Feces | 35°42'31" | 128°0'21" | PLREKRRKR*GLF | EPI_ISL_19884278 |
|  |  | Duck | A/duck/Korea/D044/2025(H5N1) | 2025-01-17 | JN | 24G1 | Organ | 35°17'27" | 127°0'21" | PLREKRRKR*GLF | EPI_ISL_19884279 |
|  |  | Duck | A/duck/Korea/D051/2025(H5N1) | 2025-01-21 | GB | 24G4 | Organ | 36°18'47" | 128°17'58" | PLREKRRKR*GLF | EPI_ISL_19884280 |
|  |  | Duck | A/duck/Korea/D058/2025(H5N1) | 2025-01-24 | CB | 24G1 | Swab | 36°53'0" | 127°29'52" | PLREKRRKR*GLF | EPI_ISL_19884281 |
|  |  | Chicken | A/chicken/Korea/C061/2025(H5N1) | 2025-01-30 | JB | 24G1 | Organ | 35°51'35" | 126°56'1" | PLREKRRKR*GLF | EPI_ISL_19884301 |
|  |  | Duck | A/duck/Korea/D063/2025(H5N1) | 2025-01-31 | JB | 24G1 | Organ | 35°44'56" | 126°40'26" | PLREKRRKR*GLF | EPI_ISL_19884282 |
|  |  | Duck | A/duck/Korea/D064/2025(H5N1) | 2025-02-01 | JN | 24G1 | Organ | 35°6'9" | 126°29'19" | PLREKRRKR*GLF | EPI_ISL_19884283 |
|  |  | Chicken | A/chicken/Korea/C071/2025(H5N1) | 2025-02-04 | CB | 24G1 | Organ | 36°52'24" | 127°30'24" | PLREKRRKR*GLF | EPI_ISL_19884302 |
|  |  | Chicken | A/chicken/Korea/C090/2025(H5N1) | 2025-02-07 | JB | 24G1 | Organ | 35°59'43" | 126°49'51" | PLREKRRKR*GLF | EPI_ISL_19884303 |
|  |  | Chicken | A/chicken/Korea/C095/2025(H5N1) | 2025-02-09 | JB | 24G1 | Organ | 35°50'49" | 126°55'12" | PLREKRRKR*GLF | EPI_ISL_19884304 |
|  |  | Duck | A/duck/Korea/D135/2025(H5N1) | 2025-03-04 | JN | 24G1 | Organ | 35°18'13" | 126°33'25" | PLREKRRKR*GLF | EPI_ISL_19884284 |
|  |  | Chicken | A/chicken/Korea/C161/2025(H5N1) | 2025-03-08 | CN | 24G1 | Organ | 36°44'12" | 127°7'41" | PLREKRRKR*GLF | EPI_ISL_19884305 |
|  |  | Chicken | A/chicken/Korea/C194/2025(H5N1) | 2025-03-19 | SJ | 24G1 | Swab/Organ | 36°41'26" | 127°10'26" | PLREKRRKR*GLF | EPI_ISL_19884306 |
|  |  | Chicken | A/chicken/Korea/C195/2025(H5N1) | 2025-03-19 | CN | 24G1 | Organ | 36°43'41" | 127°8'47" | PLREKRRKR*GLF | EPI_ISL_19884307 |
|  |  | Chicken | A/chicken/Korea/C198/2025(H5N1) | 2025-03-20 | CB | 24G1 | Organ | 36°35'38" | 127°19'44" | PLREKRRKR*GLF | EPI_ISL_19884308 |
|  |  | Chicken | A/chicken/Korea/C199/2025(H5N1) | 2025-03-21 | SJ | 24G1 | Swab/Organ | 36°43'20" | 127°10'58" | PLREKRRKR*GLF | EPI_ISL_19884309 |
|  |  | Chicken | A/chicken/Korea/C200/2025(H5N1) | 2025-03-24 | SJ | 24G1 | Swab/Organ/Feces | 36°41'53" | 127°9'20'' | PLREKRRKR*GLF | EPI_ISL_19906989 |
|  |  | Chicken | A/chicken/Korea/C201/2025(H5N1) | 2025-03-25 | CN | 24G1 | Organ | 36°45'6'' | 127°6'56'' | PLREKRRKR*GLF | EPI_ISL_19906990 |
|  |  | Chicken | A/chicken/Korea/C214/2025(H5N1) | 2025-04-03 | CN | 24G1 | Organ | 36°42'22'' | 127°15'40'' | PLREKRRKR*GLF | EPI_ISL_19906991 |
|  |  | Chicken | A/chicken/Korea/C215/2025(H5N1) | 2025-04-04 | CN | 24G1 | Organ | 36°50'14'' | 127°4'57'' | PLREKRRKR*GLF | EPI_ISL_19906992 |
|  |  | Chicken | A/duck/Korea/D216/2025(H5N1) | 2025-04-04 | CB | 24G1 | Swab/Feces | 36°44'26'' | 127°29'52'' | PLREKRRKR*GLF | EPI_ISL_19906993 |
|  |  | Chicken | A/chicken/Korea/C268/2025(H5N1) | 2025-04-19 | CN | 24G1 | Organ | 36°52'36'' | 127°1'10'' | PLREKRRKR*GLF | EPI_ISL_19906994 |

JB, Jeonbuk; JN, Jeonnam; GG, Gyeonggi; CB, Chungbuk; CN, Chungnam; SJ, Sejong; GW, Gangwon; GN, Gyungnam; GB, Gyungbuk; IC, Incheon

Table S2. Nucleotide sequences identities (%) between genes of a novel H5N3 HPAIV(WF369-1/2024) genes and previously reported H5N3 HPAIV viruses

| Korean H5N3 HPAIV(WF369-1/2024)  VS. other H5N3 HPAIVs | Nucleotide sequences identities (%) | | | | | | | | GISAID  no. | Reference No. |
| --- | --- | --- | --- | --- | --- | --- | --- | --- | --- | --- |
|  | PB2 | PB1 | PA | HA | NP | NA | M | NS |  |  |
| A/chicken/Taiwan/01174/2015(H5N3) | 94.04 | 93.01 | 90.94 | 92.96 | 93.52 | 96.45 | 91.14 | 92.96 | EPI_ISL_219873 | 38 |
| A/goose/Taiwan/01038/2015(H5N3) | 94.04 | 93.01 | 90.85 | 92.96 | 93.45 | 96.38 | 91.24 | 92.72 | EPI_ISL_219872 | 38 |
| A/chicken/Taiwan/a174/2015(H5N3) | 94.04 | 93.1 | 90.99 | 92.96 | 93.45 | 96.52 | 91.14 | 92.96 | EPI_ISL_179025 | 37 |
| A/red_knot/Germany-SH/AI03424/2020(H5N3) | 90.7 | 96.26 | 95.64 | 98.65 | 92.59 | 91.49 | 98.88 | 97.85 | EPI_ISL_1205489 | 35 |
| A/curlew/France/PPNL-21P003648/2021(H5N3) | 90.79 | 95.95 | 95.45 | 98.71 | 92.65 | 91.35 | 98.88 | 97.61 | EPI_ISL_18718171 | 36 |
| A/red_knot/France/PPNL-21P003249/2021(H5N3) | 90.83 | 96 | 95.45 | 98.77 | 92.59 | 91.42 | 98.98 | 97.73 | EPI_ISL_18718061 | 36 |
| A/red_knot/France/PPNL-21P005932/2021(H5N3) | 90.83 | 96.04 | 95.54 | 98.65 | 92.38 | 91.42 | 99.08 | 97.97 | EPI_ISL_18718063 | 36 |
